# Host cysteine proteases promote the severity of catheter-associated urinary tract infection and kidney fibrosis

**DOI:** 10.1128/mbio.02161-25

**Published:** 2025-09-22

**Authors:** Wei Xu, Jian Chen, Lisa K. McLellan, Ana L. Flores-Mireles, David A. Hunstad, Michael G. Caparon

**Affiliations:** 1 Department of Molecular Microbiology, Washington University School of Medicine 12275 , Saint Louis, Missouri, USA; 2 Center for Women’s Infectious Disease Research, Washington University School of Medicine, Saint Louis, Missouri, USA; 3 Department of Biomedical Sciences, Joan C. Edwards School of Medicine, Marshall University 4034, Huntington, West Virginia, USA; 4 Department of Genetics, Washington University School of Medicine 258944 , Saint Louis, Missouri, USA; 5 Department of Pediatrics, Washington University School of Medicine 12275 , Saint Louis, Missouri, USA; University of California, Berkeley, California, USA

**Keywords:** *Enterococcus faecalis*, cystene protease, E64, inflammation, catheter-associated urinary tract infection (CAUTI), kidney fibrosis, cathepsins, eosinophils

## Abstract

The bacterium *Enterococcus faecalis* is a leading cause of catheter-associated urinary tract infection (CAUTI), whose treatment is increasingly challenged by antibiotic resistance. In examining alternative therapies, we previously found that the cysteine protease inhibitor E64 dramatically reduced bladder inflammation and bacterial dissemination in a murine model of *E. faecalis* CAUTI. However, the role of cysteine proteases in pathogenesis and the target(s) of E64 are unknown. Here, we found that while it did not affect *E. faecalis* growth *in vitro*, in a murine CAUTI model, E64 reduced host caspase-1-dependent cellular apoptosis and necrosis, levels of several pro-inflammatory cytokines, bladder epithelial damage, and formation of renal abscesses. Analysis of host cell transcription and inflammatory cell populations revealed that E64 did not affect neutrophil numbers but did enhance the expression of C-C chemokine receptor type 3 (CCR3), a receptor for eosinophil-specific chemokines, with a concomitant increase in eosinophil numbers. Treatments that reduced or increased the eosinophil response (anti-interleukin 5 antibody or eotaxin, respectively) confirmed a role for eosinophils in controlling bacterial burdens. Analysis of a panel of host cysteine proteases *in vivo* demonstrated that E64 decreased the activation of cathepsin L; subsequently, we found that the infection of cathepsin L-deficient mice yielded lower catheter and bladder colonization compared to wild-type mice. In total, we have shown that host cysteine proteases exacerbate CAUTI pathogenesis, suggesting that cysteine protease modulation may represent a novel approach for the treatment of persistent CAUTI.

**IMPORTANCE:**

Catheter-associated urinary tract infections (CAUTIs) are the most prevalent healthcare-associated infection globally, with *Enterococcus faecalis* posing a significant threat due to widespread antibiotic resistance. This study identifies host cysteine proteases—particularly cathepsin L and caspase-1—as unrecognized drivers of CAUTI pathogenesis and renal fibrosis. Pharmacologic inhibition of these proteases using E64 reduces bladder inflammation, epithelial disruption, and kidney abscesses, while restoring fibrinogen and collagen homeostasis. Strikingly, E64 treatment unmasks a protective eosinophil response via CCR3 signaling that enhances bacterial clearance. Genetic deletion of cathepsin L recapitulates these protective effects, establishing it as a key host factor in *E. faecalis* persistence. These findings reveal host cysteine proteases as viable therapeutic targets for CAUTI and provide proof-of-concept for host-directed strategies that bypass antibiotic resistance.

## INTRODUCTION

Affecting over 2 million patients each year with 100,000 deaths and $4.5 billion in additional healthcare costs, hospital-acquired infections (HAI) are a major healthcare problem (1, 2). Catheter-associated urinary tract infections (CAUTI) are the most common HAI worldwide and account for up to 40% of HAI in the United States (2). More than 560,000 patients develop CAUTI each year, and it has become the most common source of secondary bloodstream infections (3–6). Enterococci, including *Enterococcus faecalis* and *Enterococcus faecium*, are the second most common bacterial genus causing CAUTI (6), in addition to causing uncomplicated urinary tract infections (UTI) (7), infective endocarditis (IE) (8), and bacteremia (9, 10). Complicating treatment is their inherent resistance to heat, aseptic solutions, and multiple antibiotics, including cephalosporins and monobactams. Of concern is the rising rate of acquired resistance to vancomycin, the agent commonly used to treat multidrug-resistant enterococci (11–13). Thus, there is an urgent need to understand the molecular mechanisms of CAUTI pathogenesis for the development of new, cost-effective, and antibiotic-sparing therapies.

In prior work, we showed that catheterization of the bladder leads to the deposition of host fibrinogen (Fg) onto the catheter and the bladder epithelium, which enterococci both consume as a nutrient source and use as a substrate for attachment to form catheter biofilm (14). More recently, we found that two tandemly expressed secreted proteases of *E. faecalis*, Gelatinase and Serine Protease (GelE and SprE), support the ability of *E. faecalis* to both digest Fg for growth and to form biofilm in urine (15). While GelE-SprE mutants were less virulent in a murine CAUTI model (15), we found that other proteases also contribute to disease outcome. In the absence of infection, urinary catheterization alone results in the release of Fg into the bladder along with inflammation that is independent of both glucocorticoid and neurogenic inflammation (16). By 24 h post-catheterization, bladder edema and inflammation result in the shedding of bladder epithelial cells, production of host cytokines, and infiltration of inflammatory myeloid cells. Paradoxically, this inflammatory cascade is not protective, but rather is required for the establishment of *E. faecalis* CAUTI (17, 18). In examining this sequence of events, we found that treatment of infected mice with E64, a highly specific inhibitor of papain-like cysteine proteases, improved CAUTI outcomes by reducing inflammation, bacterial burdens, and spread of enterococci to the kidney (15). Taken together, these data indicate that one or more bacterial or host cysteine proteases contribute to CAUTI pathogenesis. However, since neither SprE (a serine protease) nor GelE (a metalloprotease) is inhibited by E64, the target(s) of E64 inhibition are unknown.

In the present report, we examined how E64 treatment reduces inflammation to protect against the severity of CAUTI and associated renal damage in a murine model following catheterization and subsequent infection by *E. faecalis*. We identified direct and indirect targets of E64 and its influence in modulating innate immunity, including inhibition of proinflammatory cytokine production and recruitment of inflammatory cell populations, which revealed a novel protective role for eosinophils in CAUTI. This study illuminates how host cysteine proteases promote CAUTI pathogenesis and establishes a new strategy for therapeutic intervention for bladder infection and its sequelae.

## RESULTS

### E64 treatment reduces bladder and renal cell damage during CAUTI

**Fig 1:**
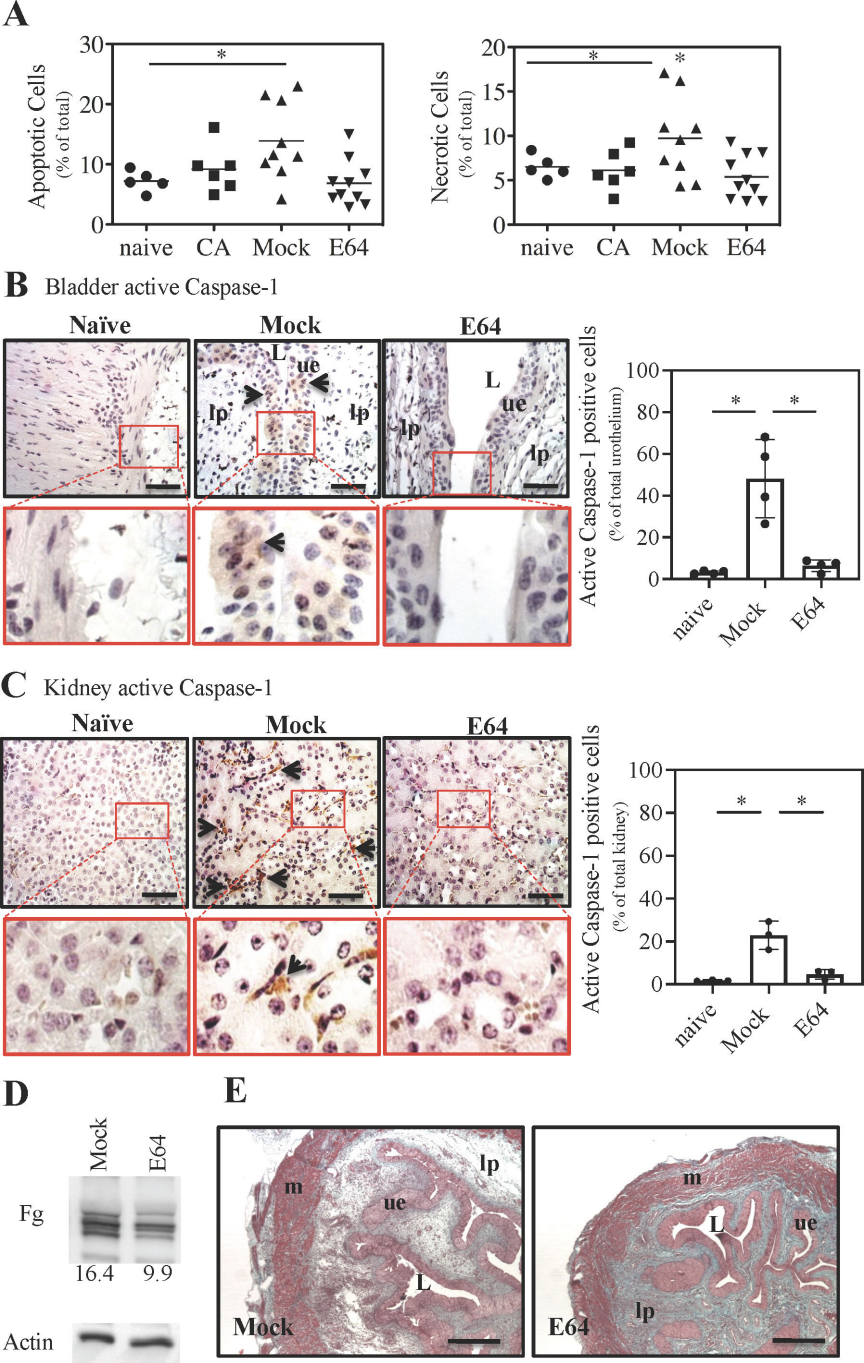
Reduced inflammation in E64-treated bladders. (**A**) Bladder single-cell suspensions prepared 2 dpi were analyzed to detect apoptosis and necrosis via flow cytometry in mice that were not catheterized and uninfected (Naive), catheterized but not infected (CA), catheterized and infected but not treated (Mock), and catheterized, infected, and treated with E64 (E64). (**B and C**) Immunohistochemistry (IHC) of bladder/kidney sections 2 dpi from mock, E64, or naïve mice stained for active caspase-1 (brown color indicated by arrows). Scale bar: 50 µm. Red boxes highlight zoomed-in areas. Quantification of active caspase-1 staining was performed using ImageJ by calculating the percentage of positively stained cells per high-power field across ≥ 3 fields per section. (**D**) Bladder tissue homogenates were subjected to a Western blot to detect Fg and β-actin as a control. The concentration of Fg in the bladder was determined by comparing the chemiluminescent signal following staining to a standard curve prepared using pure fibrinogen (0.001–20 mg/mL). Numbers below indicate mg/mL of Fg in bladder tissue. (**E**) Bladder sections taken 2 wpi were examined by Gomori trichrome stain and light microscopy. Scale bar: 400 µm. Labels are: L, lumen; lp, lamina propria; ue, urothelium; m, muscularis. All data are from ≥3 biological replicates. Statistical comparisons were performed using a two-tailed unpaired Student’s t-test. *P < 0.05. Normality was assessed using the Shapiro-Wilk test.

*In vitro*, E64 did not inhibit the growth of *E. faecalis* strain OG1RF, nor did it inhibit the functional expression of aggregation factor (Fig. S1), whose attachment to the cell wall requires sortase A, a cysteine protease known to be critical for CAUTI virulence (19, 20). Therefore, to examine the effect of E64 treatment on the bladder epithelium, mice were treated with E64 twice daily beginning 1 day prior to catheter implantation and infection and then for an additional 2 days (timeline is shown in Fig. S2). Mice were sacrificed, a MACS cell dissociator was used to gently prepare single-cell suspensions of bladder cells, and the percentages of apoptotic and necrotic cells were determined by FACS (see “Materials and Methods”). Uncatheterized and uninfected (i.e., naïve) bladders harbored a low amount of apoptotic or necrotic cells (~6% each, Fig. 1A), and implantation of a catheter in the absence of infection did not significantly alter these percentages (CA, Fig. 1A). Infection of catheter-implanted bladders that were not treated (Mock, Fig. 1A) significantly increased the proportion of apoptotic (~14%) and necrotic (~10%) cells, while E64 treatment of CAUTI bladders reduced apoptotic and necrotic cell percentages to levels equivalent to naïve bladders (E64, Fig. 1A).

To examine how E64 was inhibiting cellular death, paraffin-embedded sections of bladders from 2 days post-infection (dpi) were subjected to immunostaining to detect the active form and overall levels of the pro-apoptotic cysteine protease caspase-1. Bladders and kidneys from mock-treated CAUTI mice displayed pronounced staining for active caspase-1 that was not apparent in either CAUTI mice treated with E64 or naïve mice (arrows, Fig. 1B and C). Only a limited increase in staining for total caspase-1 levels was apparent for infected vs. naïve mice (Fig. S3), suggesting that the activation of existing pro-caspase-1, rather than its enhanced expression, is responsible for the apoptosis phenotype in CAUTI mice. Expression of other cysteine proteases was also examined. These include (i) caspase-3, known as an executor of apoptosis (21), (ii) cathepsin L (CTSL) and cathepsin B (CTSB), which are lysosomal proteases responsible for protein turnover, antigen processing, and extracellular matrix remodeling (22), and (iii) CC motif chemokine receptor (CCR3), a chemokine receptor that regulates the recruitment and activation of eosinophils (23). These results are summarized in Table S1. Caspase-3 showed a mild increase in expression in mock-treated bladders but was unchanged in kidneys. CCR3 expression was selectively increased in E64-treated bladders, suggesting a potential role in modulating the local immune response. In the kidney, IL-1α was decreased with E64 treatment, and CTSL was increased in mock-treated animals, with evidence of the mature form, while CTSB levels remained unchanged across all conditions.

### E64 reduces deposition of Fg and collagen in the bladder and kidney

**Fig 2:**
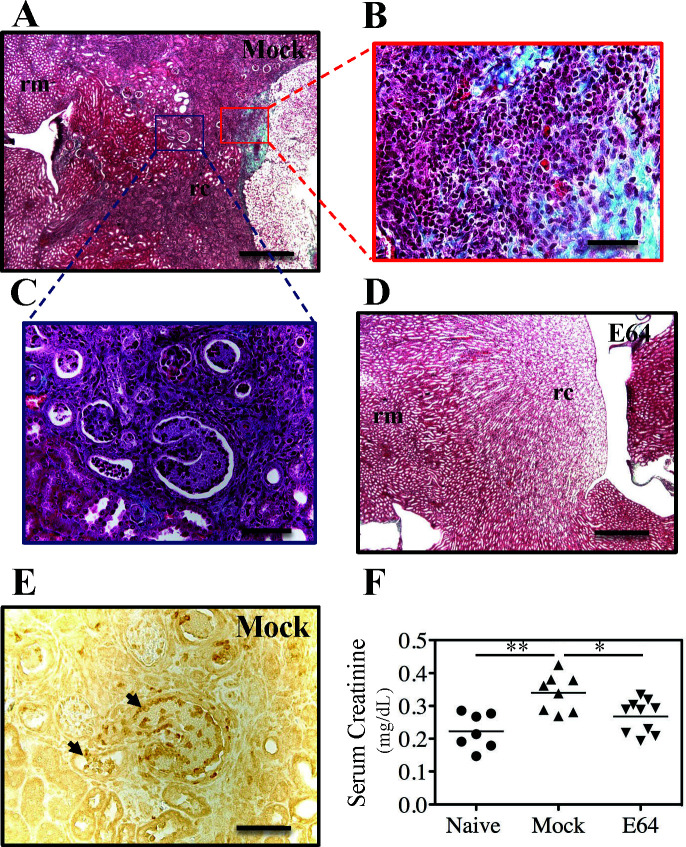
Reduced renal damage in E64-treated kidney. Paraffin-embedded kidney sections 2 wpi from mock (**A–C**) and E64 (**D**) mice were examined by Gomori trichrome staining and light microscopy. Areas of necrotic abscess in the renal cortex are outlined in (**A**) and enlarged in (**B**) and (**C**), as indicated. Collagen deposition appears green. (**E**) Immunohistochemistry with anti-*E*. *faecalis* (*Streptococcus* Group D antigen, brown color at arrows). The section shown is the subsequent tissue section of (**C**). (**F**) Serum creatinine levels 2 wpi; mean and SEM are shown. Labels are: rm, renal medulla; rc, renal cortex. Scale bar in (**A**) and (**D**): 400 µm. Scale bar in (**B**), (**C**), and (**E**): 50 µm. All data are from ≥3 biological replicates. Statistical comparisons were performed using a two-tailed unpaired Student’s t-test. * *P* < 0.05, ** *P* < 0.001. Normality was assessed using the Shapiro-Wilk test.

We have shown that the deposition of Fg into the catheterized bladder is a critical component of CAUTI pathogenesis, where it promotes growth and attachment of *E. faecalis* to catheters for the formation of biofilm (14). Upon catheterization, the concentration of Fg in the bladder increases to 8.0 from the 1.0 mg/mL found in the naïve murine bladder (24), as determined by a standard curve of Fg. Infection of catheter-implanted bladders increased Fg concentrations to over 16 mg/mL, while treatment with E64 reduced Fg concentrations to 9.9 mg/mL (Fig. 1D). To further examine the effects of E64, Mock vs E64 bladders were histologically compared at 2 weeks post-infection (wpi). Mock-treated CAUTI bladders exhibited significant edema, and Gomori trichrome staining revealed a pronounced loss of collagen as compared to E64-treated CAUTI bladders (Fig. 1E). While most mice resolve bacteriuria over time, at 2 wpi, 10%–20% of Mock mice developed renal scars with evidence of bacterial infiltration. Scarring was apparent in Gomori trichrome-stained kidney sections with extensive abscess formation in cortical and medullary locations, reflected by dense collagen deposition and retraction of cortical tissues (Fig. 2A). The surrounding renal parenchyma was also damaged, showing interstitial infiltration, glomerular sclerosis, thickened tubular membranes, and evidence of fibrosis (Fig. 2B and C). These findings were largely absent in E64-treated mice (Fig. 2D). As shown by staining with an anti-*E*. *faecalis* antibody, bacterial antigens were present in the tubules of mock-treated CAUTI mice (Fig. 2E), and these mice had a 57% increase in serum creatinine, a biomarker of acute kidney injury (25), which was significantly reduced in E64-treated and infected mice (Fig. 2F).

### Systemic alterations by E64

**Fig 3:**
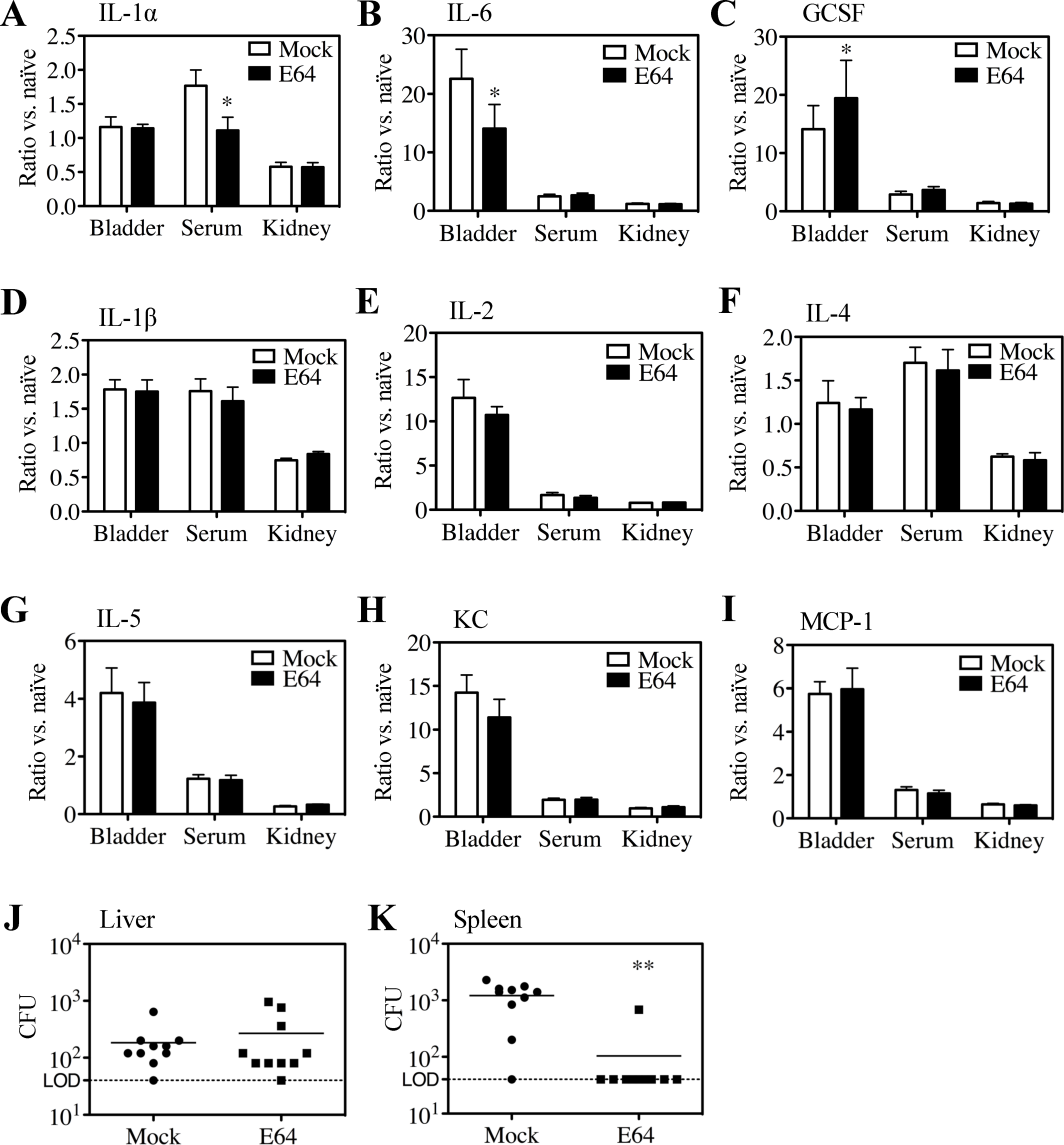
Inflammatory signaling and dissemination are altered in E64-treated mice. (**A–I**) Levels of the indicated cytokines were determined in serum and in homogenates from bladder and kidney prepared from mock and E64 mice 2 dpi. Cytokine levels are presented as the fold change relative to naïve mice, where naïve mice are assigned a level of 1.0. Mean and SEM are shown. Total CFU recovered 2 dpi from homogenates of (**J**) liver and (**K**) spleen. Each symbol represents an individual mouse, and symbols touching the dashed lines indicate values below the limit of detection (LOD, 40 CFU). Horizontal lines indicate mean values. All data are from ≥3 biological replicates. Statistical comparisons were performed using a two-tailed unpaired Student’s t-test. * *P* < 0.05, ** *P* < 0.001. Normality was assessed using the Shapiro-Wilk test.

To examine how E64 treatment may alter cytokine responses, a multianalyte analysis of serum and homogenized bladders and kidneys was performed 2 days post-infection (dpi). Mock-treated CAUTI mice displayed significant increases in bladder IL-6 and G-CSF, with a moderate increase in serum IL-1α as compared to naïve mice, consistent with a prior report (26); these were significantly reduced in E64-treated mice (Fig. 3A through C). Levels of IL-1α in bladders were not significantly impacted by catheterization, infection, or E64 treatment (Fig. 3A); however, E64 treatment of CAUTI mice was associated with higher levels of G-CSF (Fig. 3C). Bladder IL-1β, IL-2, IL-4, IL-5, KC, and MCP-1 in mock-treated CAUTI mice were all significantly increased over naïve mice; however, these analytes were not altered by E64 treatment (Fig. 3D through I). In examining systemic bacterial burdens, colony-forming units (CFU) in the liver did not differ between E64- and mock-treated CAUTI mice 2 dpi (Fig. 3J). In contrast, while almost all mock-treated CAUTI mice exhibited bacterial spread to the spleen, all but one of the E64-treated CAUTI mice had no detectable spleen titers (Fig. 3K). These data show that E64 treatment alters a subset of cytokines associated with CAUTI and inhibits dissemination from the urinary tract to the spleen.

### E64 treatment promotes expression of CCR3 in the bladder

**Fig 4:**
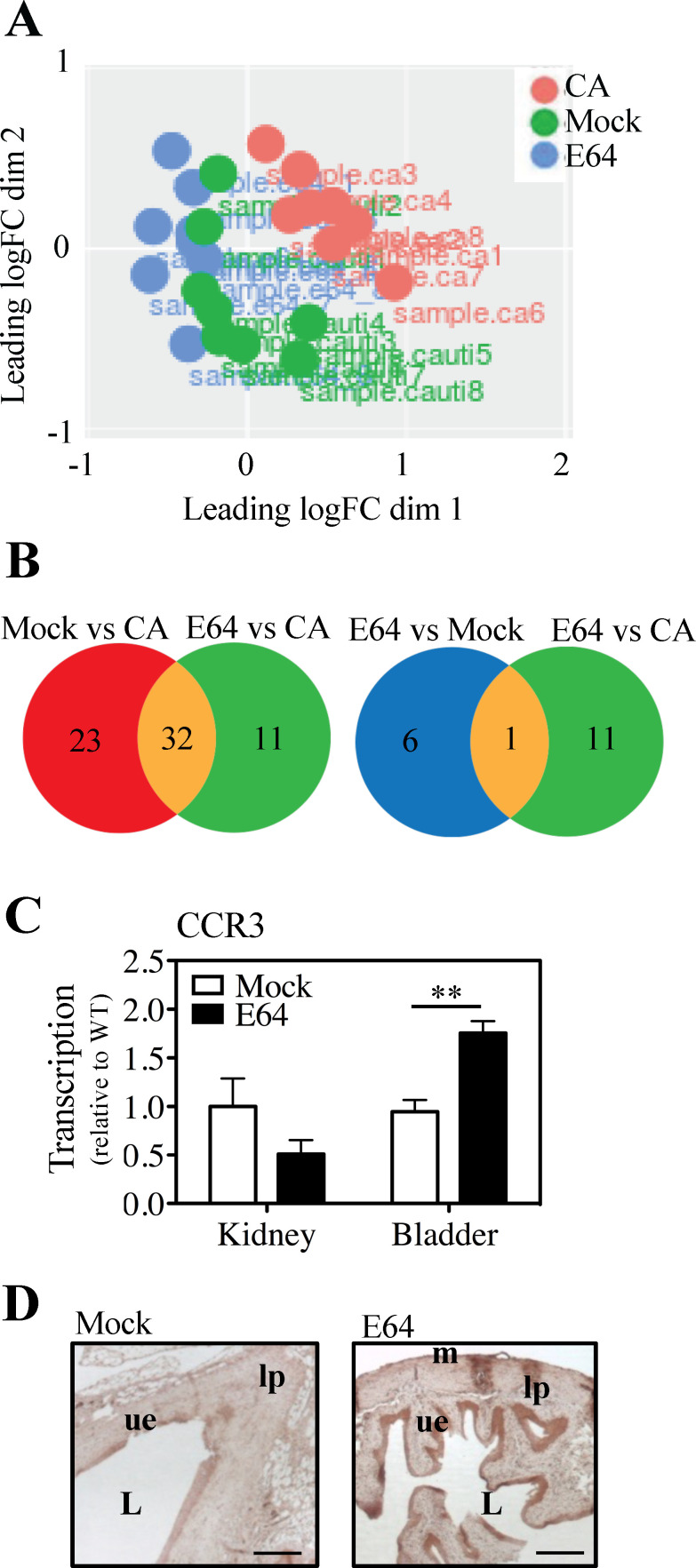
CCR3 is upregulated in E64-treated bladders. Mice were catheterized (CA), catheterized and infected with *E. faecalis*, and treated with phosphate-buffered saline (PBS; Mock) or E64. RNA from mouse bladders 2 dpi was used for RNA-seq and RT-PCR. (**A**) Dissimilarity measurement on the leading LogFC RNA across all samples was coordinated in a two-dimensional MDS plot. Each dot represents data from a single mouse bladder. (**B**) Venn diagram of genes upregulated > 1 LogFC across multiple samples. (**C**) Relative transcription (to WT) of the gene-encoding mouse CCR3 was determined by real-time RT-PCR. Mean and SEM are shown. (**D**) Immunohistochemistry (IHC) of bladder sections 2 dpi from mock- and E64-treated CAUTI mice stained for anti-CCR3 (brown). Scale bar: 50 µm. Labels are: L, lumen; lp, lamina propria; ue, urothelium; m, muscularis. All data are from ≥3 biological replicates. Statistical comparisons were performed using a two-tailed unpaired Student’s t-test. **P < 0.001. Normality was assessed using the Shapiro-Wilk test.

To better understand how E64 affects host responses and how this affects susceptibility to *E. faecalis* CAUTI, a global RNA-seq analysis was conducted on the bladder of catheterized mice that were (i) not infected (Catheterized Alone, CA); (ii) infected and Mock treated (Mock); or (iii) infected and treated with E64 (E64). Principal component analysis indicated significant overlap in gene expression profiles in the two groups of infected mice (Mock and E64; green and blue circles, respectively; Fig. 4A), which were distinct from uninfected mice (CA, red circles; Fig. 4A). To identify genes associated with E64’s ability to reduce inflammation, approx. 6,000 detectable and annotated genes were examined for upregulation by infection (≥2.0-fold, *P* < 0.05) in the Mock- or E64-treated CAUTI mice as compared to CA mice. The Mock vs CA comparison revealed 55 upregulated genes specific to infection, and the E64 vs CA comparison included 43 genes; 32 upregulated genes were common to these two sets (Fig. 4B). Most of these common genes are related to immune cell proliferation to promote bacterial defense (Table S2). Direct comparison of the two CAUTI groups (E64 to Mock) revealed seven genes upregulated specifically with E64 treatment (Table 1), of which one was also found in the E64-to-CA comparison (Fig. 4B). Annotated as the eosinophil marker C-C chemokine receptor type 3 (CCR3), its upregulation upon E64 treatment was confirmed by RT-PCR and immunohistochemical analyses of bladder tissue (Fig. 4C and D). Increased expression was specific to the bladder, as CCR3 expression did not differ between E64- and mock-treated kidneys (Fig. 4C).

**TABLE 1:** Log2 FC in Gene Expression in E64- vs Mock-Treated CAUTI Bladders

Genes	Description	logFC	*P* value
Ccr3	C-C chemokine receptor type 3	1.28	4.90E-03
Kcnn3	Small conductance calcium-activated potassium channel protein 3	1.11	1.09E-06
Prss35	Inactive serine protease 35	1.06	2.96E-06
Col19a1	Collagen alpha-1 chain	1.02	4.92E-08
OtogI	Otogelin-like protein	1.02	9.41E-08
Hcn1	Potassium/sodium hyperpolarization-activated cyclic nucleotide-gated channel 1	1.01	5.19E-08
Mc5r	Melanocortin five receptor	1.01	1.88E-04

Other upregulated genes suggest additional pathways contributing to E64’s therapeutic effects. For example, Kcnn3 (potassium calcium-activated channel subfamily N member 3) regulates smooth muscle excitability and neuronal signaling (27, 28). Prss35 (protease serine 35) encodes a protease implicated in extracellular matrix remodeling and tissue repair (29). Col19a1 (collagen type XIX alpha 1) is a minor collagen involved in basement membrane integrity (30). To evaluate whether E64 induces coordinated host signaling rather than broad immunosuppression, we performed Reactome pathway enrichment analysis of the RNA-seq data set (Table S3). Sixteen pathways were significantly enriched (FDR < 0.1), despite modest fold changes in individual genes. These included pathways linked to extracellular matrix organization (e.g., collagen biosynthesis and degradation), potassium and calcium-activated ion channels, and G-protein-coupled receptor (GPCR) signaling. Of note, no genes were specifically downregulated by E64 across all comparisons (Fig. S4, Table S4). This analysis indicates that E64 has a highly focused, rather than global, impact on the host response to catheterization and infection of the bladder.

### E64 promotes a protective eosinophil response

**Fig 5:**
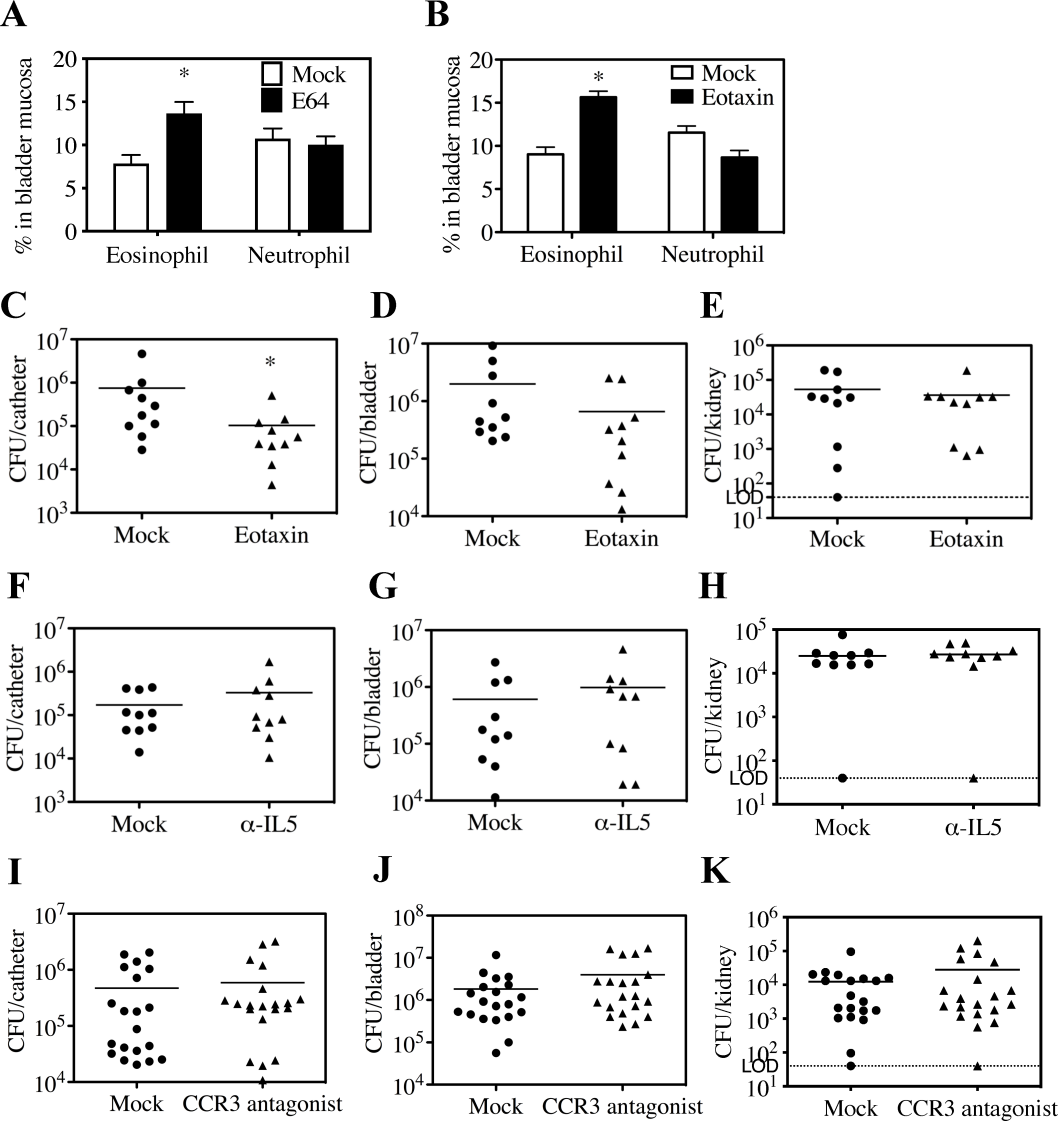
Eosinophils play a protective role in CAUTI. At 2 dpi, single cells were isolated from the bladder mucosa of CAUTI mice. The percentages of eosinophils and neutrophils were measured by flow cytometry to assess the effects of E64 treatment (**A**) or eotaxin (**B**) as compared to mock. Total CFU recovered 2 dpi were quantified for catheter, bladder, and kidney for treatment by eotaxin (**C–E**), by a-IL-5 (**F–H**), or by CCR3 antagonist SB328437 (**I–K**). Each symbol represents an individual mouse, and symbols touching the dashed lines indicate values below the limit of detection (LOD, 40 CFU). Horizontal lines indicate mean values; error bars represent SEM. All data are from ≥3 biological replicates. Statistical comparisons were performed using a two-tailed unpaired Student’s t-test. *P < 0.05. Normality was assessed using the Shapiro-Wilk test.

As CCR3 is a marker specific to eosinophils (31), we hypothesized that its enhanced expression in response to E64 correlates with elevated infiltration of eosinophils into bladder tissue. To test this, single-cell suspensions from bladder tissue were analyzed by FACS to quantify eosinophil, neutrophil, and other cell populations. Representative gating strategies are shown in Fig. S5 and S6. Our prior analysis indicated that myeloid cells (CD11b^+^) accounted for ~30% of the live cell population in CA mice at 24 h post-implantation, with neutrophils (CD11b^+^, Ly6G^hi^, Ly6C^low^) being among the most abundant immune cells (16). Here, we find that by 2 dpi, eosinophil (CD11b^+^, CD193^+^, Siglec-F^+^) numbers were similar to neutrophils in mock-treated CAUTI mice (Mock, Fig. 5A). In contrast, consistent with elevated CCR3 expression, eosinophil numbers in E64-treated CAUTI mice were increased significantly by ~60% compared to Mock, while neutrophil numbers were unaffected by E64 treatment (E64, Fig. 5A). Other immune cell populations, including T cells, monocytes, dendritic cells, and macrophages, were not significantly altered by E64 treatment (Fig. S7). To test whether an increase in eosinophils correlates with a higher degree of bacterial clearance, bladders were instilled with a single dose of the eosinophil chemoattractant eotaxin at the time of catheter implantation. Treatment with eotaxin reproduced the effect of E64 in CAUTI mice, resulting in a 60% increase in eosinophil numbers in treated mice relative to Mock-treated controls; neutrophil numbers were unaffected by eotaxin treatment (Fig. 5B). CAUTI was less severe in eotaxin-treated mice, with lower bacterial burdens on catheters and bladders, although no significant difference in bacterial burdens was observed in kidneys (Fig. 5C through E). Conversely, treatment of CAUTI mice with a monoclonal antibody to neutralize IL-5, a key mediator of eosinophil activation (32), resulted in a 70% reduction in bladder infiltrating eosinophils (Fig. S8) and no change in bacterial burdens on catheters, bladders, or kidneys (Fig. 5F through H). Treatment with the highly selective CCR3 antagonist SB328437 (33) had a similar effect as the anti-IL-5 antibody (Fig. 5I through K).

### E64 inhibition of cathepsin L improves infection outcomes in CAUTI

**Fig 6:**
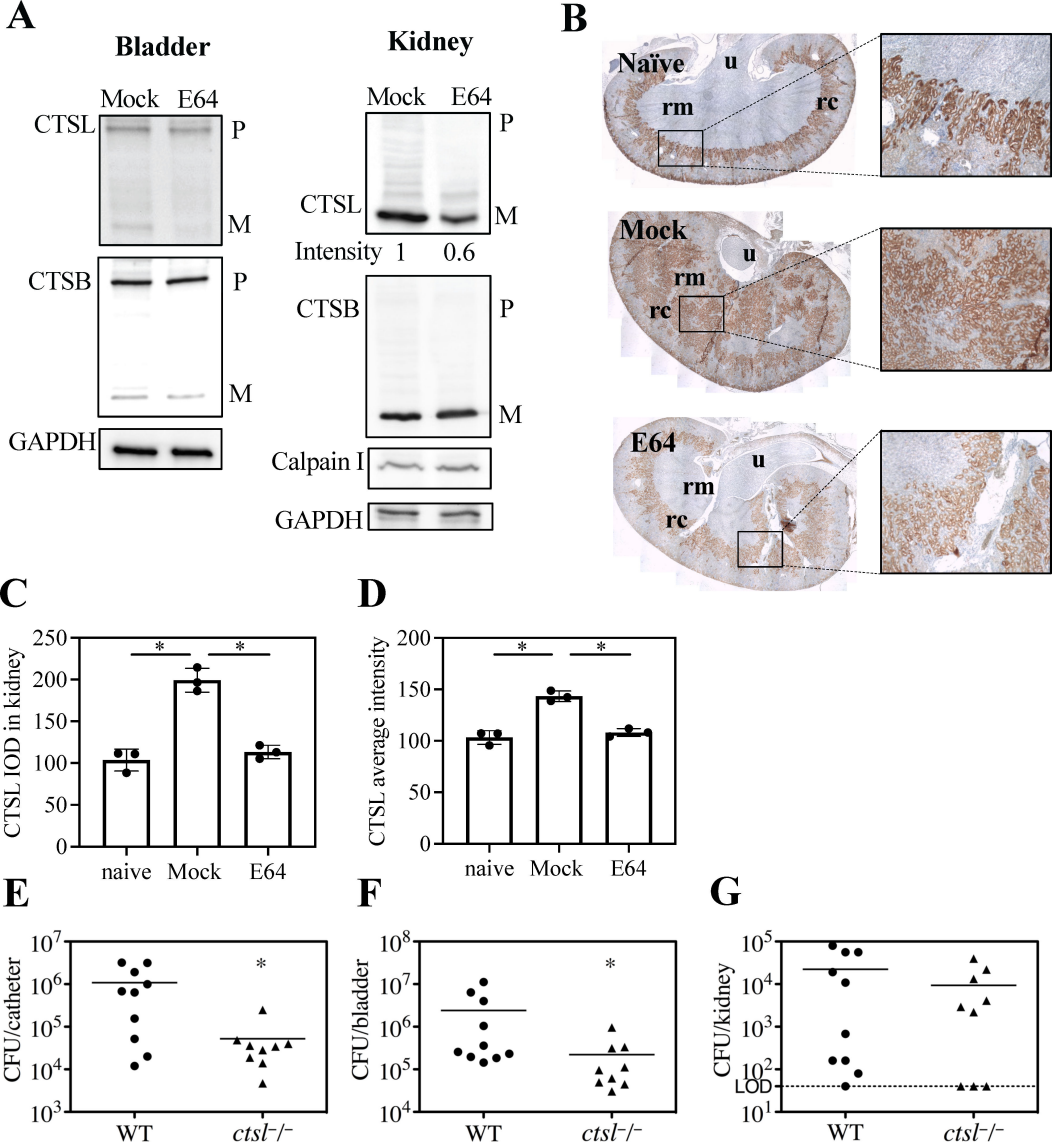
CTSL promotes infection by *E. faecalis*. (**A**) Bladder and kidney tissue homogenates from mock- or E64-treated CAUTI mice were subjected to Western blot for CTSL, CTSB, calpain I, and GAPDH as a control. P: precursor; M: mature. The band intensity was measured using ImageJ. (**B**) Immunohistochemistry (IHC) of whole kidney sections 2 dpi to detect CTSL (brown) in naïve mice and in mock or E64-treated CAUTI mice. Black boxes highlight zoomed-in areas. (**C and D**) Quantification of CTSL staining in the kidney by integrated optical density (IOD) and average intensity. (**E–G**) CAUTI in *ctsl^-/-^* vs WT mice was compared by quantifying CFU recovered from the catheter, bladder, and kidney. Each symbol represents an individual mouse, and symbols touching the dashed lines indicate values below the limit of detection (LOD, 40 CFU). Horizontal lines indicate mean values. Labels are: rm, renal medulla; rc, renal cortex; u, ureter. All data are from ≥ 3 biological replicates. Statistical comparisons were performed using a two-tailed unpaired Student’s t-test. *P < 0.05. Normality was assessed using the Shapiro-Wilk test.

To look for direct targets of E64, we compared expression and activation of several proteases in bladder and kidney tissue from mock- or E64-treated CAUTI mice. Western blot analysis of tissue extracts of bladder and kidney from E64-treated CAUTI mice revealed reduced activation of the cysteine protease cathepsin-L (CTSL), but not cathepsin-B (CTSB) or calpain 1 (Fig. 6A). Focusing on CTSL, we observed that its expression in Naïve mice was predominantly at the corticomedullary junction and segment S3 of the cortex (proximal tubules) (Naïve, Fig. 6B through D). Enterococcal CAUTI elicited patchy but substantial CTSL expression in the medulla, presumably reflecting tubular/cellular injury (Mock, Fig. 6B through D). In contrast, E64 treatment of infected mice reduced medullary CTSL expression to a level resembling that in Naïve mice (E64, Fig. 6B through D). To examine the influence of CTSL on pathogenesis, *ctsl*^-/-^ mice were examined in the CAUTI model. While these mice can display periodic hair loss, skin thickening, reduced serum levels of glucose, and altered expression of Foxp3 in CD4^+^ T cells, they are fertile and have no defects in renal development or function (34–36). This analysis revealed significant attenuation in bacterial burdens from catheters and bladder tissue 2 dpi compared to WT mice, while kidney CFUs were not statistically different in *ctsl*^-/-^ mice (Fig. 6E through G). These results suggest that CTSL contributes to colonization at mucosal sites such as the bladder and catheter. In contrast, kidney colonization may reflect downstream effects of ascending infection and anatomical variation and thus be less directly influenced by CTSL activity.

## DISCUSSION

In pursuing alternative, antibiotic-sparing therapies for CAUTI, we previously found that the cysteine protease inhibitor E64 reduced histologic bladder inflammation and bacterial dissemination in a murine model of *E. faecalis* CAUTI (15). In the present study, we examined possible E64 targets and found that inhibition of several host cysteine proteases, including caspase-1 and cathepsin L, significantly attenuated the severity of experimental CAUTI by limiting cell death and inflammation, enhancing expression of protective cytokines, reducing kidney damage, and promoting a beneficial eosinophil response (Table S1). The low toxicity of E64 and other protease inhibitors developed for the treatment of infectious disease (37, 38) suggests that inhibition of host cysteine proteases may represent an adjunct approach for the treatment of CAUTI.

To better understand the immunomodulatory effects underlying these therapeutic benefits, we examined how E64 impacts cytokine production during infection. Our observed reduction in pro-inflammatory cytokines (e.g., IL-1α, IL-6) and G-CSF in E64-treated mice (Fig. 3) highlights the immunomodulatory potential of cysteine protease inhibition during CAUTI. While the cellular origin of these cytokines remains unresolved in this study, prior work in urinary tract infections implicates urothelial cells, macrophages, and neutrophils as key contributors to cytokine production during infection (39). For example, IL-6 and G-CSF are classically secreted by epithelial cells and myeloid cells in response to bacterial sensing (18, 40, 41), while IL-1α can be released by damaged urothelium or infiltrating macrophages (42). E64’s broad inhibition of cysteine proteases may suppress cytokine release across multiple cell types. Future studies employing cell-specific protease inhibition (e.g., conditional knockout models) or single-cell transcriptional profiling of infected tissues could delineate whether E64 preferentially targets urothelial, immune, or stromal populations to attenuate inflammation.

An important finding was that the E64-induced eosinophil response was associated with decreased bacterial burdens on implanted catheters and decreased systemic dissemination. Neutrophils are the most abundant inflammatory cells recruited into the bladder during CAUTI and are important in the control of CAUTI, as neutropenic mice experience significantly more severe CAUTI (16). However, neutrophils themselves are not sufficient to clear the infection (16). The reason for this failure is unknown but may involve the ability of *E. faecalis* to form biofilm on catheters (16).

In contrast, the increased eosinophil response induced by E64 may reflect a more regulated or reparative antibacterial mechanism, potentially limiting neutrophil-mediated tissue damage. How eosinophils contribute to improving the CAUTI outcomes observed here is also unknown, as the exact roles of eosinophils in inflammatory disease are controversial. In fact, eosinophils have typically been associated with adverse outcomes in allergic disease and found to contribute to inflammatory tissue damage (43), such that the therapeutic use of agents that prevent eosinophil accumulation at sites of inflammation has been proposed (44, 45). On the contrary, eosinophil secretion of anti-inflammatory mediators, including TGFβ1^25^ and protectin D1 (PD1), may limit inflammatory responses to exert a protective effect, as observed in acute murine colitis, asthma, and acute peritonitis (46–48). PD-1 can reduce levels of inflammatory cytokines, including TNFα, IL-1β, IL-6, and iNOS (47), suggesting that an increase in eosinophil numbers may act to reduce inflammation and tissue damage induced by catheterization and infection, consistent with our observations here of eotaxin effects during CAUTI.

The mechanism of eosinophil-mediated CAUTI protection could also include direct action on pathogen viability. For example, secretory products from eosinophils can directly inhibit the growth of *Plasmodium falciparum* (49) and contribute to host defense against viral pathogens, including HIV (50). Eosinophil-derived granule mediators such as eosinophil cationic protein (ECP), eosinophil peroxidase (EPO), and major basic protein 1 (MBP-1) can kill and digest intracellular bacteria (51), which may contribute to killing of *Staphylococcus aureus*, *Escherichia coli*, and *Listeria monocytogenes* (52, 53). Extracellular bacteria can be killed by eosinophils through the release of mitochondrial DNA-containing “traps” into the extracellular space to engulf bacteria in an instantaneous catapult-like fashion (54). How eosinophils specifically promote improved outcomes in CAUTI remains to be determined. However, the observation that protective eosinophil responses are suppressed in the absence of E64 treatment indicates that *E. faecalis* exploits the role of host cysteine proteases in regulating the balance between protective and non-protective eosinophil functions.

The observed increase in eosinophil recruitment with E64 treatment may reflect protease-dependent regulation of chemokine signaling or immune cell crosstalk. For example, E64 may stabilize eosinophil-attracting chemokines such as eotaxins by preventing their degradation. Alternatively, reduced neutrophil-driven inflammation may relieve the suppression of eosinophil trafficking. These possibilities are not mutually exclusive and merit further investigation. Functional studies (e.g., eosinophil depletion via anti-Siglec-F antibodies or genetic models) are needed to confirm eosinophil necessity in E64-mediated protection. Future studies comparing eosinophil antimicrobial activity *in vitro* (±E64) or their impact on epithelial cells (±E64) could clarify whether protease inhibition directly enhances eosinophil recruitment or function.

E64 is an epoxide that can irreversibly inhibit a broad range of cysteine proteases by forming a covalent adduct with the active-site cysteine (37). Due to its low toxicity, it has been employed therapeutically to inhibit host and pathogen cysteine proteases in a variety of infection models encompassing bacterial (55–57), fungal (58), and parasitic (59) infections, and for multiple viruses (60, 61), including SARS-CoV-2 (62, 63). There are several possible host targets of E64, including caspases, cathepsins, and calpains, which all have well-documented regulatory roles in the proinflammatory response, including proteolytic activation of IL-1β, a cytokine upregulated during experimental *E. faecalis* CAUTI (26). E64 can inhibit cysteine proteases involved in cellular apoptosis and can inhibit the cathepsin family of proteases found in lysosomes that are involved in the terminal steps of both necrosis and apoptotic cell death (64, 65). While the observed ~8% absolute increase in apoptotic cells (6% vs. 14%) was statistically significant, its modest magnitude suggests that apoptosis alone may not fully account for the observed pathology. Protease-mediated mechanisms such as necrosis or disruption of epithelial barrier integrity may synergize with apoptosis to amplify tissue injury. E64’s broad inhibition of cysteine proteases involved in both apoptosis and necrosis likely contributes to its therapeutic effect by attenuating multiple injury pathways. Numerous other cellular processes are regulated by host cysteine proteases, including epithelial cell membrane rupture, tight-junction integrity, and inflammatory signaling (66). These represent possible mechanisms through which host cysteine proteases could exacerbate the pathogenesis of CAUTI and contribute to the release of Fg into the bladder lumen. Understanding the molecular targets inhibited by E64 in CAUTI will be important for the development of highly specific inhibitors targeting the host proteases that contribute to the catheter-induced pathological changes in the bladder that promote CAUTI pathogenesis.

The observed reduction in caspase-1 activation and bladder cell death suggests that E64 may suppress components of inflammasome signaling and pyroptosis (67, 68). Since caspase-1 is a cysteine protease, it is plausible that E64 interferes with its proteolytic activity, thereby limiting inflammatory cell death. Future studies are needed to directly evaluate inflammasome activation and pyroptosis in this model.

This study also shows that beyond its therapeutic benefit in the bladder, E64 can also limit renal destruction and bacterial dissemination to other organs through inhibition of host cysteine proteases such as CTSL. Interestingly, CTSL expression is increased in many glomerular diseases, such as membranous glomerulonephritis, minimal change disease, and focal segmental glomerulosclerosis (69). CTSL-mediated degradation of proteins essential for normal podocyte architecture can result in proteinuria and renal failure and accompanies diabetic nephropathy (70). Enhanced medullary expression of CTSL during *E. faecalis* CAUTI likely contributed to tubular/cellular injury to promote dissemination and increased kidney bacterial burdens, which were reduced in both E64-treated and *ctsl*^-/-^ mice. Caspase-3 activation in endothelial cells can trigger extracellular CTSL release, resulting in the cleavage of extracellular matrix components (71). Since E64 can inhibit caspases, protection of the extracellular matrix from CTSL activity represents an additional mechanism for E64-mediated protection from enterococcal dissemination and kidney damage and suggests that the interaction between CTSL and caspases deserves further investigation. Future studies employing tissue-specific deletion of CTSL in bladder epithelium or myeloid cells, coupled with direct measurement of CTSL activity in bladder tissue during infection (with or without E64 treatment), could refine our mechanistic understanding of how CTSL contributes to catheter-associated bladder inflammation and bacterial persistence.

The observed reduction in kidney fibrosis following E64 treatment is a clinically relevant outcome that likely stems from modulation of the host response, as E64 does not directly inhibit *E. faecalis*. While the precise mechanism remains to be defined, one possibility is that cysteine protease activity contributes to fibrogenesis by promoting tissue injury and sustained inflammation, which, in turn, activates fibrotic pathways such as TGF-β signaling or fibroblast activation (72, 73). Inhibiting these proteases may suppress the release of fibrogenic mediators from damaged epithelial or immune cells. Alternatively, reduced fibrosis may be an indirect consequence of attenuated bacterial dissemination and inflammation, rather than a direct action on fibrotic pathways. Future studies using *in vitro* co-culture models, such as exposing renal epithelial or fibroblast cells to *E. faecalis* with or without E64, could help clarify whether E64 limits fibrosis by dampening epithelial damage, immune cell activation, or fibroblast signaling. Delineating these mechanisms will be important to establish whether host cysteine protease inhibition offers organ-protective benefits beyond infection control.

Through an analysis of how the cysteine protease inhibitor E64 improves infection outcomes in a murine model of *E. faecalis* CAUTI, this study has identified potential targets for the development of antibiotic-sparing therapeutics for the treatment of CAUTI caused by multidrug-resistant uropathogens. Advantages to this approach include that a host-directed therapeutic will not be under selection pressure for the development of resistance and that protease inhibitors have an established record for efficacy and safety in the treatment of a range of diseases. This study has also shown how a specific uropathogen can exploit how host proteases regulate inflammation to promote CAUTI.

## MATERIALS AND METHODS

### Antibodies

Antibodies against caspase-1 (Abcam, ab1872), Active caspase-1 (Cell Signaling Technology, D57A2), cleaved caspase-3 (Cell Signaling Technology, D3E9), IL-1α (Chemcon, AB1414), calpain I (Abcam, ab28257), CTSB (LifeSpan Bioscience, B3313), CTSL (Abcam, ab133641), CCR3 (Abcam, ab32512), Fg (Sigma, F8512), and *Streptococcus* Group D antigen (14) were used for Western blot or immunohistochemistry (IHC). Antibodies against CD11b (BD Pharmingen, 557657), CD193 (BioLegend, 144505), and Ly6G/Ly6C (BioLegend, 108429) were used for FACS.

### Bacterial strain and growth conditions

Experiments involving *E. faecalis* used strain OG1RF (74) that was regularly maintained on Brain Heart Infusion (BHI; BD, Franklin Lakes, NJ) agar plates supplemented with 25 µg/mL of rifampin and 25 µg/mL of fusidic acid (75, 76). Unless otherwise specified, cultures were inoculated from a single colony into BHI broth and grown at 37°C for 18 h. Bacteria were grown under anaerobic conditions in sealed jars using a commercial gas generator (GasPak, BBL) (77). Bacterial titers were determined as previously described (14). To assess the effect of E64 on *E. faecalis* growth, overnight cultures of OG1RF were diluted 1:100 into fresh BHI broth containing either vehicle (phosphate-buffered saline [PBS]) or E64. Cultures were incubated at 37°C in 96-well plates. Aliquots were serially diluted and plated on BHI agar to determine CFUs.

### Mouse catheter implantation and infection

Six-week-old female wild-type C57BL/6Ncr mice were purchased directly from Charles River Laboratories, and *ctsl^+/+^*, *ctsl^–/–^* (Cat L-008352) mice were purchased from Jackson Laboratories and bred in-house. Mice were transurethrally implanted with a 5 mm length of platinum-cured silicone catheter as previously described (26). Where indicated, mice were transurethrally infected immediately after implantation with a dose of ~2 × 10^7^ CFU in PBS in a total volume of 50 µL. For drug therapy, E64 (Roche, 10874523001), anti-mouse IL-5 (BD Pharmingen, 554393), SB 328437 (CCR3 antagonist, Sigma, 247580-43-4), and mouse eotaxin (R&D systems, 420-ME) were dissolved in PBS according to the manufacturers’ recommendations to achieve the concentration stated in the text. For E64, anti-mouse IL-5, and SB 328437 treatment, mice received intraperitoneal injections of 100 µL of the solution or PBS alone (Mock) and were implanted and infected. Eotaxin was instilled into the bladder directly via the urethra during catheter implantation. The schedule of E64, anti-mouse IL-5, and eotaxin treatment and infection is shown in Fig. S2. At the indicated times, mice were sacrificed to aseptically harvest bladders, kidneys, liver, and spleens. Homogenates were prepared in 1 mL sterile PBS using a FastPrep-24 5G homogenizer (MP Biomedicals) and plated on BHI supplemented with rifampin and fusidic acid (25 µg/mL of each). The bacterial burden in each sample was calculated as CFU/organ. A 500 µL aliquot of each bladder and kidney homogenate was centrifuged at 13,000 rpm in a benchtop microcentrifuge for 5 min at 4°C, and supernatants were removed and stored at −80°C for cytokine analysis.

### Histological analyses, microscopy, and image processing

For histological analyses, bladders were fixed in 10% formalin for 24 h and dehydrated in 70% ethanol overnight at 4°C. They were then embedded in paraffin, sectioned, and stained with hematoxylin and eosin (H&E) or Gomori trichrome stain for light microscopy. IHC analyses of potential E64 targets were performed using the SignalStain IHC kit (Cell Signaling Technology, cat. #8114) according to the manufacturer’s protocol. IHC analyses of *E. faecalis* were performed as described previously (78) using an anti-*Streptococcus* Group D antigen antibody. Immunofluorescence staining and microscopy were performed as described previously (14). Images were acquired using a Leica DM1000 microscope equipped with an EC3 digital camera. The histological images presented were stitched together from multiple overlapping images using the Photomerge function of Adobe Illustrator CC (ver. 2017.1.0) or LAS EZ software (Leica Microsystems). For publication, images were processed using Adobe Photoshop CC (ver. 2017.1.1) and prepared using Adobe Illustrator CC (ver. 2017.1.0).

### Flow cytometry

To prepare single-cell suspensions, bladders were aseptically removed and placed in ice-cold PBS. Whole bladders were inverted and mechanically dissociated using a GentleMACS dissociator in C Tubes (cat. #130-096-334, Miltenyi Biotec) according to the manufacturer’s protocol (79). To remove debris, cell suspensions were filtered through a 40 µm cell strainer (Corning Falcon, 352340). Apoptosis and necrosis in cell populations were measured using the Apoptosis/Necrosis Detection Kit (Abcam, ab176750) according to the manufacturer’s protocol. Briefly, dissociated total bladder cells were labeled with three fluorescent probes: (i) a red fluorescent phosphatidylserine (PS) sensor (Ex/Em = 630/660 nm) to detect apoptosis (PS exposure on outer membrane leaflets), (ii) DNA Nuclear Green DCS1 (Ex/Em = 490/525 nm) to identify late apoptosis/necrosis (membrane rupture permitting nuclear dye uptake), and (iii) CytoCalcein Violet 450 (Ex/Em = 405/450 nm) to label viable cells. For antibody staining (80), single-cell suspensions were preincubated with anti-CD16/CD32 Fc Block antibody (BioXCell, BE0307) in PBS for 10 min at RT before staining with the following antibodies. Antibodies were obtained from BioLegend and included: FITC anti-CD45.2 (109806), BV605 anti-MHC II (107639), PB anti-Ly6C (128014), and PE anti-mouse CCR3 (144505). The following anti-mouse antibodies were obtained from Tonbo Biosciences: PE anti-F4/80 (50-4801-U100), PercpCy5.5 anti-Ly6G (65-1276-U025), APC-Cy7 anti-CD11c (25-0114-U025), and PE-Cy7 anti-CD11b (60-0112-U100). Alexa Fluor 700 anti-CD3ε (56-0033-82) and anti-Mo CD170 (Siglec F, 414-1702-80) were from Invitrogen. Validation information for these antibodies is available at vendor websites. Cells were stained for 20 min at 4°C, washed, and fixed in 4% methanol-free paraformaldehyde (Electron Microscopy Sciences) in PBS for 20 min at 4°C. Flow cytometry data were acquired on an Agilent Technologies Novocyte and analyzed using FlowJo software (TreeStar, version 10.8.1). Gating strategies are depicted in Fig. S5 and S6.

### Western blot analyses

Tissue homogenates prepared as described above were mixed with an equal volume of 2× SDS sample buffer and subjected to immunoblotting using the primary antibodies described above and secondary goat anti-rabbit IgG conjugated with HRP (cat. #12-348, Sigma-Aldrich) at a final dilution of 1:2,000. Blots were developed and imaged using a ChemiDoc PM imaging system (Bio-Rad), as previously described (81).

### Creatinine assay

Microtainer serum separation tubes (BD, cat. #REF365967) were used to collect blood from mice at the time of sacrifice, which was then subjected to centrifugation at 15,000 × *g* for 5 min at room temperature. Serum was collected and deproteinized using a spin filter with a 10 kD molecular weight cut-off. Creatinine levels were measured using the Creatinine Assay Kit (Sigma-Aldrich, cat. #MAK080) according to the manufacturer’s instructions.

### Cytokine/chemokine analysis

Tissue homogenates or serum supernatants were thawed on ice, centrifuged at 4°C to remove any remaining particulates, and used for measurement of cytokine and chemokine expression using the Bio-Plex-Pro Mouse Cytokine 23-Plex Panel multiplex cytokine bead kit (Bio-Rad, cat. #10014942) according to the manufacturer’s instructions.

### RNA-seq and real-time RT-PCR

Total RNA from bladders and kidneys was isolated using RNeasy Plus Mini Kit (Qiagen, cat #74134) per the manufacturer’s recommended protocol. Integrity of RNA was determined using a 4200 Tapestation or an Agilent Bioanalyzer. Library preparation was performed using 10 μg of total RNA with a Bioanalyzer RIN score greater than 8.0. Ribosomal RNA (rRNA) was depleted using Oligo-dT beads (mRNA Direct kit, Life Technologies) through poly-A selection. The resulting mRNA was fragmented in a buffer containing 40 mM Tris acetate, pH 8.2, 100 mM potassium acetate, and 30 mM magnesium acetate by heating to 94°C for 150 s and then reverse transcribed to cDNA with random hexamers and SuperScript III RT polymerase (Life Technologies, cat #18080093) per the manufacturer’s instructions. A second-strand reaction was performed to yield double-stranded cDNA, which was blunt-ended; an A base was added to 3′ ends, followed by ligation of Illumina sequencing adapters to the ends. The resulting fragments underwent 12 cycles of amplification using primers that integrated unique index tags. Subsequently, the amplified products were sequenced on an Illumina HiSeq-3000 platform, generating single reads extending 50 bases. Reads were aligned using Ensembl (release 76) (82) with top-level assembly performed using STAR (version 2.0.4b (83). Relationships between experimental groups were illustrated by Venn diagrams generated with Eulerr 4.1.0 (https://jolars.github.io/eulerr/). RT-PCR analyses of CCR3 expression were performed with iScript cDNA synthesis kit (Biorad, 1708890 and the primer pairs #1 “F-TCCATGCAATGCTGATGCTC” and “R-AATTGTCAACTGGCCAGCAC,” and the primer pairs #2 “F-ATCGTCCATGCTGTGTTTGC” and “R-GCAGGAAAACTCTCCAAAGCTG” (84). Relative transcript abundance was determined by the –DDC_T_ method normalized to GAPDH expression, determined using the primer pair #1 “F-GATTTGGCCGTATTGGGCGC” and “R-TTAGTGGGGTCTCGCTCCTG,” and primer pair #2 “F-CACAGTCCATGCCATCACTG” and “R-CGGCACGTCAGATCCACGAC” as described (84).

### Statistical analyses

Data are derived from at least three independent experiments, with the values presented representing the mean ± SEM for each group. The difference between WT and mutant strains was tested for significance using a two-tailed Mann-Whitney U test available in GraphPad Prism software, where *, **, and *** indicate *P* < 0.01, < 0.05, and <0.001, respectively. For all tests, the null hypothesis was rejected for *P* > 0.05. Values below the limit of detection (LOD) for CFU assays were assigned the LOD value (40 CFU).

## Data Availability

Raw data files for RNASeq are deposited on NCBI with BioProject ID PRJNA1109797 and are publicly available.

## SUPPLEMENTAL MATERIAL

Supplemental Figures mbio.02161-25-s0001.pdf Figures S1-S8.

Table S1 mbio.02161-25-s0002.docx Expression level changes of potential E64 targets.

Table S2 mbio.02161-25-s0003.xlsx Genes upregulated after mock or E64 treatment.

Table S3 mbio.02161-25-s0004.xlsx The 16 most upregulated pathways.

Table S4 mbio.02161-25-s0005.xlsx Genes downregulated after mock or E64 treatment.

ASM does not own the copyrights to Supplemental Material that may be linked to, or accessed through, an article. The authors have granted ASM a non-exclusive, world-wide license to publish the Supplemental Material files. Please contact the corresponding author directly for reuse.
