# Supplementary material for: Host cysteine proteases promote the severity of catheter-associated urinary tract infection and kidney fibrosis
Source: mBio. 2025 Sep 22;16(11):e02161-25. doi: 10.1128/mbio.02161-25 (PMC12607876; doi:10.1128/mbio.02161-25)
Supplement: Supplemental Figures — Figures S1-S8. [file mbio.02161-25-s0001.pdf]

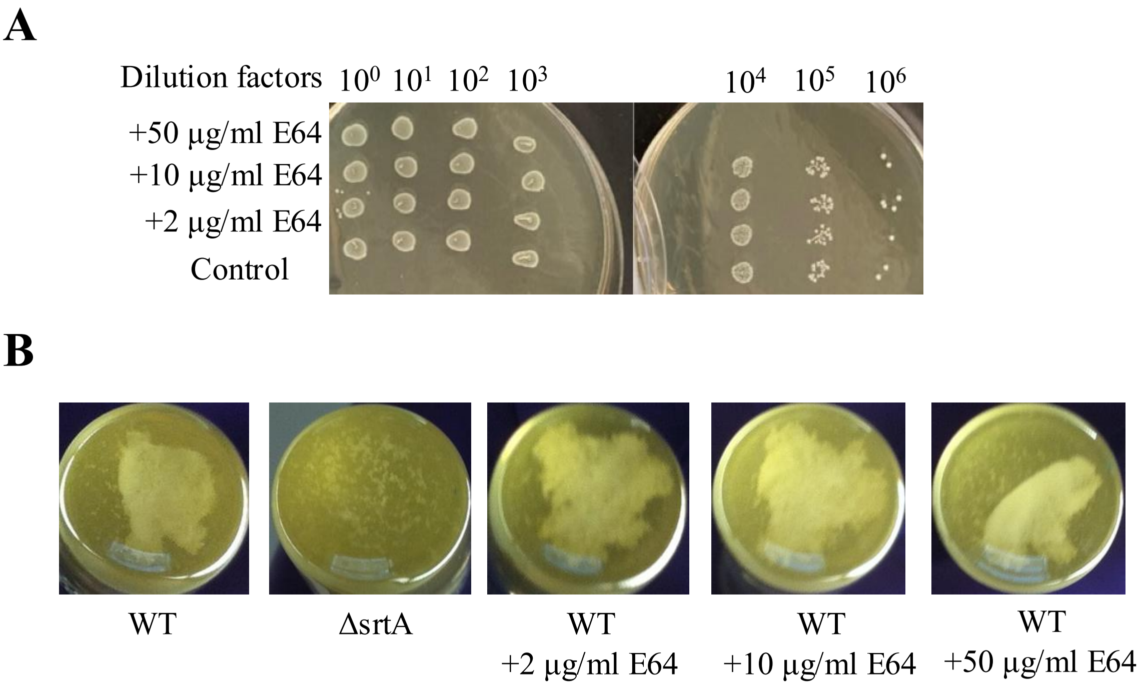

Figure S1. E64 does not inhibit growth or target sortase in *E. faecalis*. (**A**) *In vitro* growth of *E. faecalis* OG1RF in BHI broth in the absence or presence of E64. (**B**) Supernatant of strain OG1X WT was used as a source of pheromone to induce clumping. *E. faecalis* OG1SS::pCF10 WT and ΔsrtA strains were grown overnight in BHI. Cultures were then back diluted 1:10 fold in BHI and 0.25 ml of OG1X filter-sterilized supernatant was added with shaking at 100 RPM at 37°C for 2 hrs. Various concentrations of E64 were added at the same time of dilution. Clumping of bacteria were visualized at the bottom of tubes. All data are from ≥3 biological replicates.

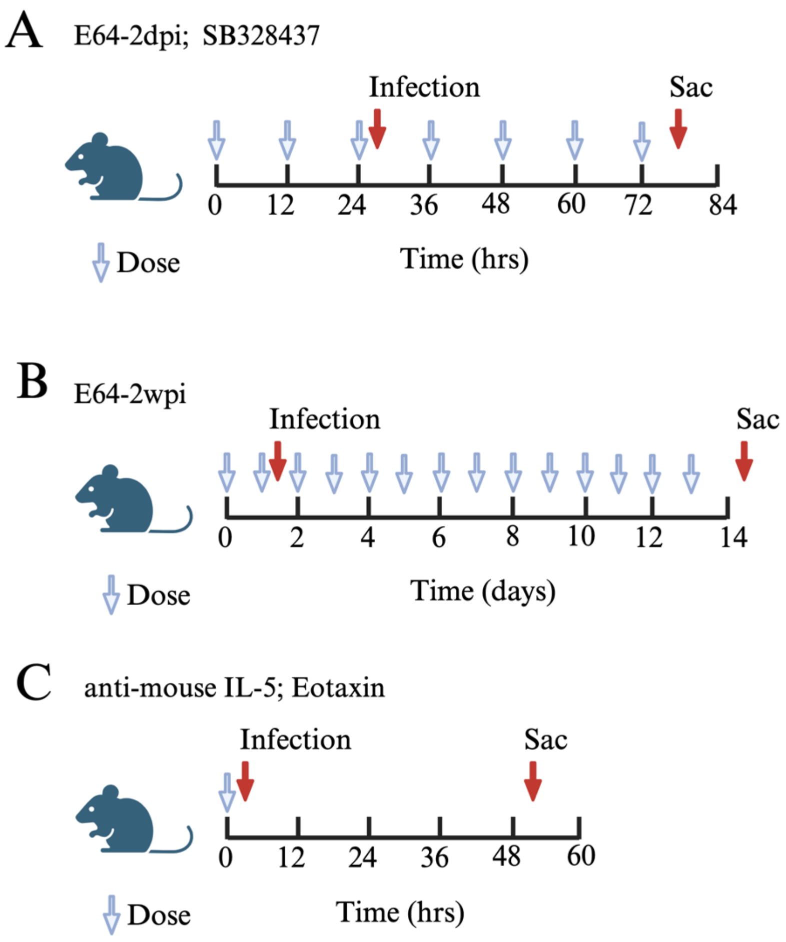

Figure S2. Experimental timeline for treatment of murine CAUTI. Groups of mice were treated with the drugs indicated or mock-treated (PBS), infected and analyzed for determination of bacterial burdens as shown. For the arrows representing dose, E64, anti-mouse IL-5 and SB 328437 were administered by intraperitoneal injection and eotaxin was administered by direct injection into urethra. Inhibitors were used at the following concentrations: E64: 2 mg/kg/day; anti-mouse IL-5: 2 mg/kg/dose; SB 348437: 2 mg/kg/day; Eotaxin: 0.08 mg/kg/dose. Infection: time of catheter implantation followed immediately by infection by the WT strain. Sac: time of mouse sacrifice.

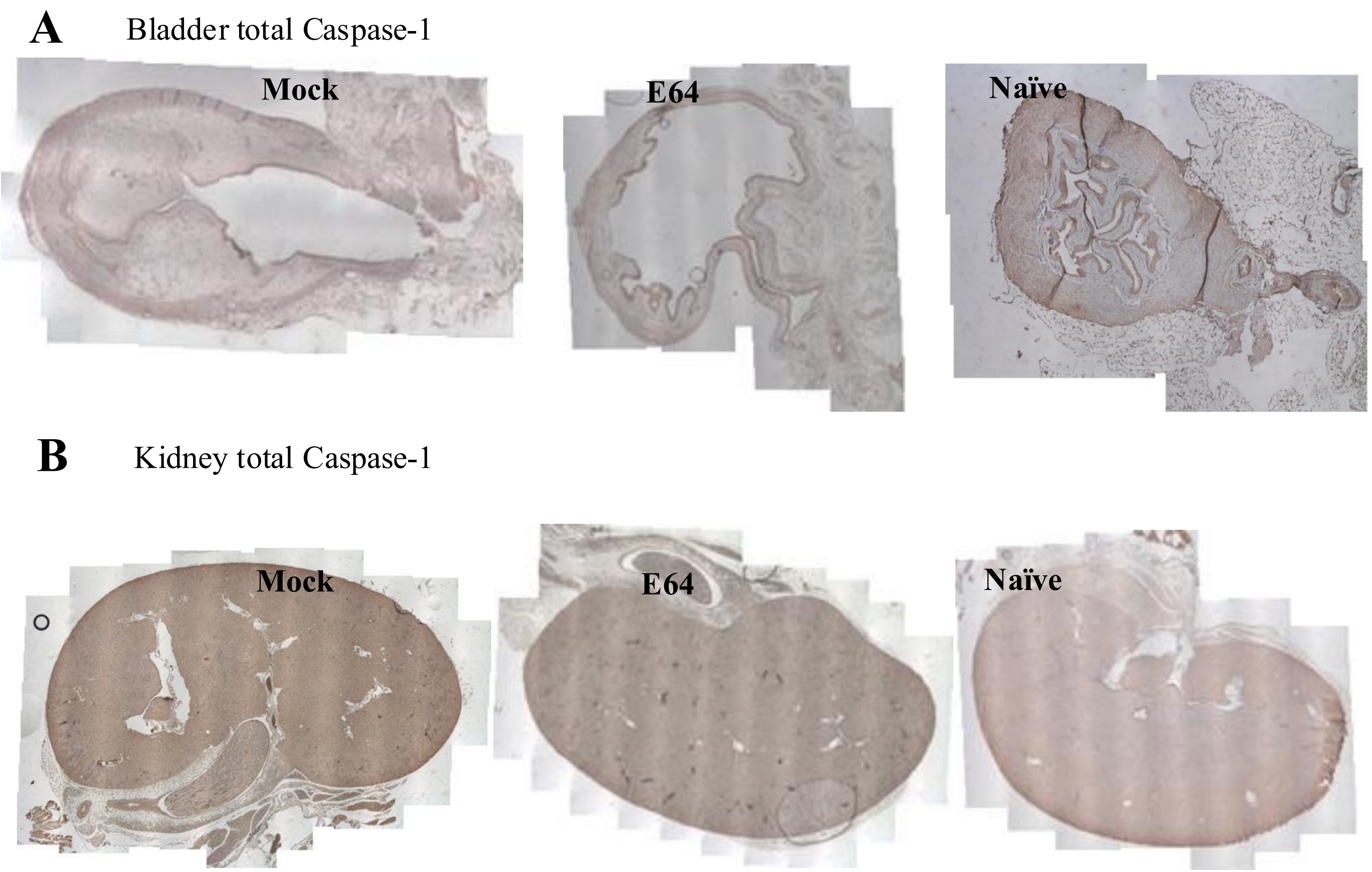

Figure S3. Immunohistochemistry (IHC) of bladder/kidney sections. At 2 dpi bladders from Mock, E64 or naïve mice were harvested and analyzed by IHC for total caspase-1, which appears as the brown color in images.

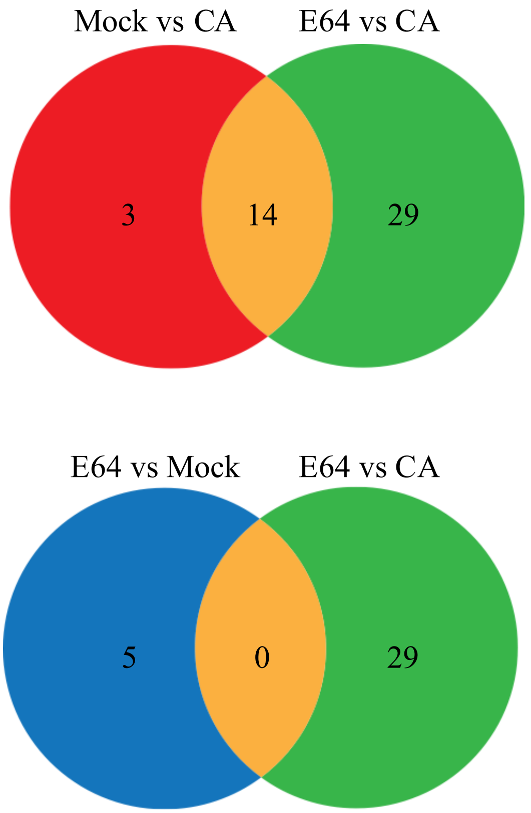

Figure S4. Genes downregulated after Mock- and E64- treatment. Mice were catheterized (CA), catheterized and infected with *E. faecalis*, and treated with PBS (Mock) or E64. Following 2 dpi, RNA was harvested for analysis by RNA-seq, and the number of genes downregulated (>2.0 LogFC) in the indicated comparisons are plotted in a Venn diagram.

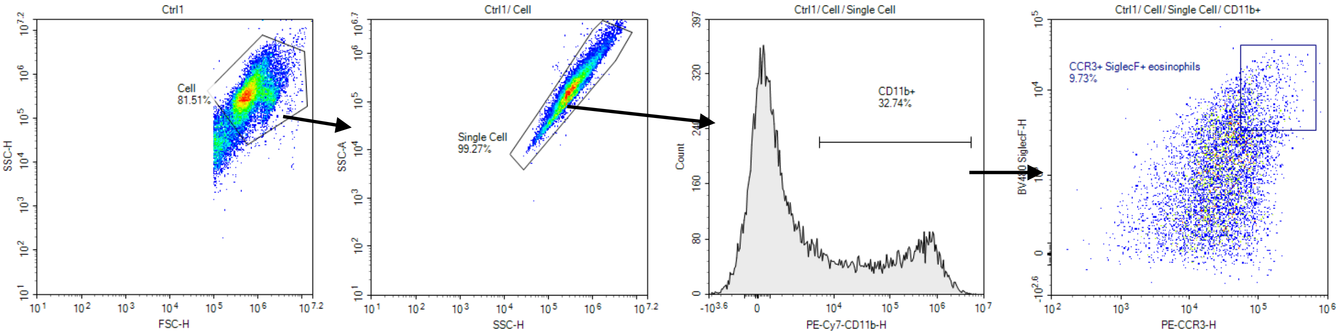

Supplementary Figure 5. FACS configuration for eosinophil population. Representative flow cytometry plots for WT *Enterococcus faecalis* infected bladder, showing the gating strategy for eosinophil populations.

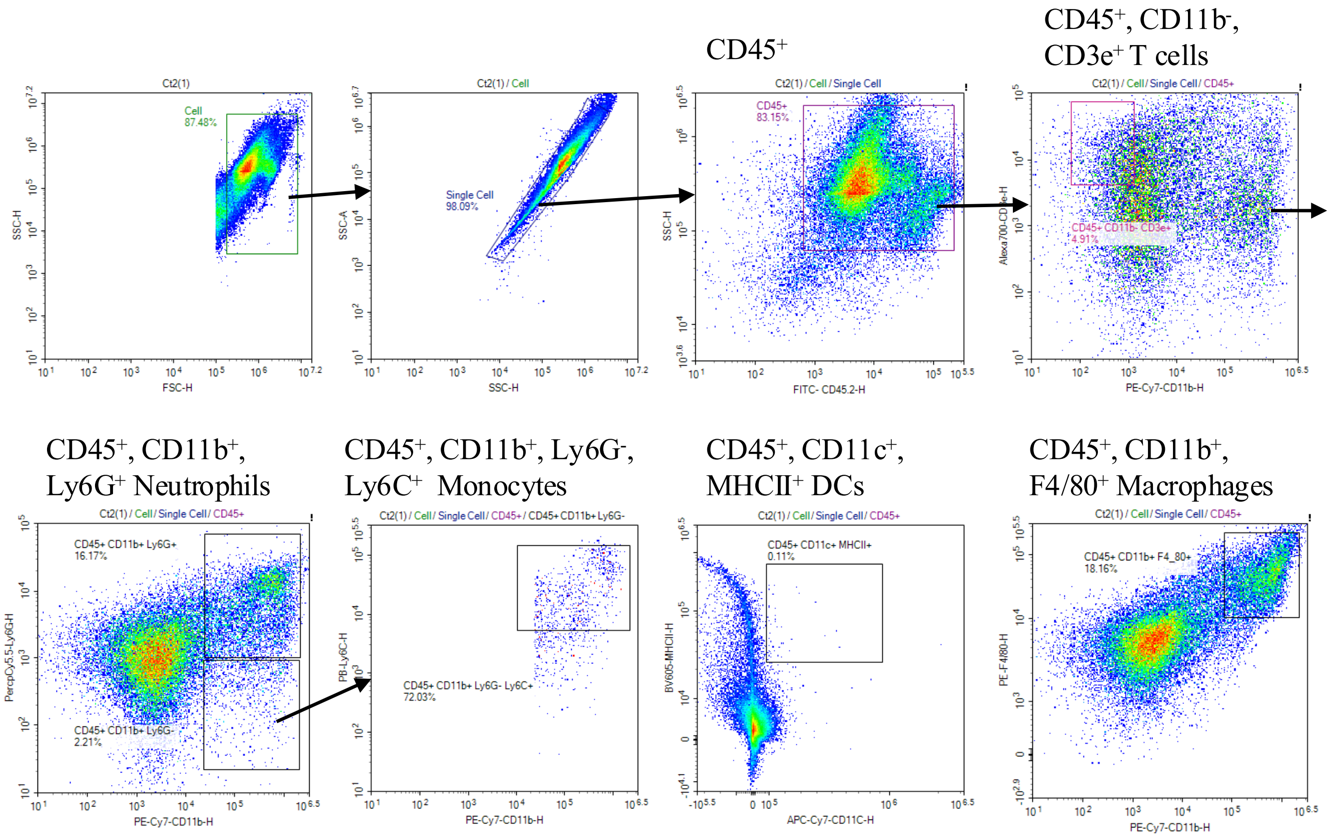

Supplementary Figure 6. FACS configuration for immune cell population. Representative flow cytometry plots for WT *Enterococcus faecalis* infected bladder, showing the gating strategy for cell populations.

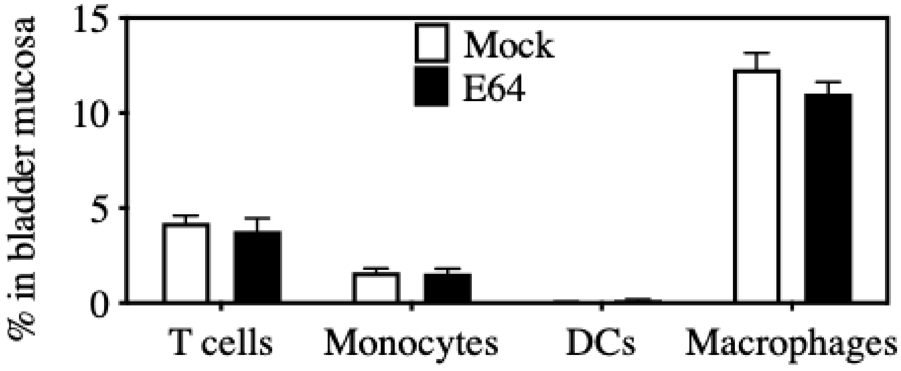

Supplementary Figure 7. Immune cell population in bladders. The distribution of specific cell types in the total $CD45^+$ population of cells at 2-dpi was evaluated by flow cytometry. DCs, dendritic cells. n = 8 mice per group, biological replicates, data presented represents the mean and SEM.

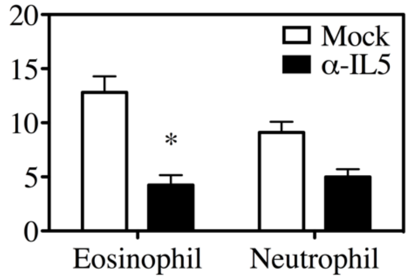

Figure S8. Eosinophils depletion by α-IL-5. At 2 dpi, single cells were isolated from the bladder mucosa of CAUTI mice. The percentages of eosinophils and neutrophils were measured by flow cytometry to assess the effects of α-IL-5 as compared to Mock. Horizontal lines indicate mean values; error bar represents SEM. * $p<0.05$.
