# Supplementary material for: Host cysteine proteases promote the severity of catheter-associated urinary tract infection and kidney fibrosis
Source: mBio. 2025 Sep 22;16(11):e02161-25. doi: 10.1128/mbio.02161-25 (PMC12607876; doi:10.1128/mbio.02161-25)
Supplement: Table S1 — Expression level changes of potential E64 targets. [file mbio.02161-25-s0002.docx]

Table S1. Expression Level Changes of Potential E64 Targets.

| Antibody against | Expression in Bladder^#^ | Expression in Kidney |
| --- | --- | --- |
| Caspase-3^^^ | Mild increase in Mock-treated | Same as naïve |
| Caspase-1^$^ | Same as naïve | Mild increase in Mock-treated |
| Active caspase-1 | Increase in Mock-treated, mild increase in E64-treated | Increase in Mock-treated, mild increase in E64-treated |
| IL-1α | Same as naïve | Decrease in E64-treated |
| CTSL | Same as naïve | Increase in Mock-treated, migration |
| CTSB | Same as naïve | Same as naïve |
| CCR3 | Increase in E64-treated | Same as naïve |

^ Caspase-3 staining is done through fluorescent 2^nd^ antibody, all the other staining were done with IHC.

# increase or decrease were compared with expression in naïve organs.

$ Caspase-1 expression is also enhanced in Mock-treated ureters.
